# High ambient temperatures associations with children and young adult injury emergency department visits in NYC

**DOI:** 10.1088/2752-5309/ace27b

**Published:** 2023-07-12

**Authors:** Blean Girma, Bian Liu, Leah H Schinasi, Jane E Clougherty, Perry E Sheffield

**Affiliations:** 1 Department of Environmental Medicine and Public Health, Icahn School of Medicine at Mount Sinai, New York, NY, United States of America; 2 Department of Population Health Science and Policy, Icahn School of Medicine at Mount Sinai, New York, NY, United States of America; 3 Department of Environmental and Occupational Health and Urban Health Collaborative, Dornsife School of Public Health, Drexel University, Philadelphia, PA, United States of America; 4 Department of Environmental and Occupational Health, Dornsife School of Public Health, Drexel University, Philadelphia, PA, United States of America

**Keywords:** extreme heat, temperature, injury, children, climate health, emergency department utilization

## Abstract

Injury is a significant health burden for children and young adult and may be an increasing concern in a warming climate. Research reveals many impacts to children’s health associated with hot weather and heatwave events, including a growing literature on the association between high ambient temperature and injury, which may vary by intent such as injury resulting from violence. However, little is known about how this association varies across different types of injury and subgroups of young people. We examined relationships between warm season ambient temperature and intentional and unintentional injury among children and young adults in New York City (NYC). Within a case-crossover design, our study observed injury-related emergency department (ED) visits from the New York Statewide Planning and Research Cooperative System administrative dataset. Injuries were categorized as unintentional or intentional injuries during the warm season (May through September) in NYC from 2005 to 2011 among patients (0, 1–4, 5–9, 10–14, 15–19, 20–25 years old (y.o.)). Conditional logistic regression models with distributed lag non-linear functions were used to model the cumulative odds ratio (OR) injury-related ED visit over 0–5 lag days. Analyses were stratified by age group and sex to understand how associations vary across young people of different age and sex. There were a total of 572 535 injury-related ED visits. The largest effect of elevated temperature (daily minimum 77°F vs 48°F) was for unintentional injury among 5–9 y.o. (OR 1.32, 95% CI 1.23, 1.42) and for intentional injury among 20–25 y.o. (OR 1.54, 95% CI 1.28, 1.85). Further stratified analyses revealed that the highest risk of unintentional injury was among 5–9 y.o. males and 20–25 y.o. males for intentional injury. Our results suggest that high ambient temperatures are associated with higher odds of unintentional and intentional injuries among children. This work adds to a growing body of literature demonstrating the adverse impacts of heat on children, and suggests the need for messaging to parents and children about adopting adaptive strategies to prevent injuries when it is hot outside.

## 1. Introduction

A growing literature on pediatric heat-health impacts has documented positive association between high outdoor temperature (heat) with various adverse health outcomes such as dehydration, heat-related illness, diarrhea and digestion disorders, infectious diseases and injury among pediatric populations (Sheffield *et al* 2018, Bernstein *et al* 2022, Uibel *et al* 2022). The health effects of heat on children are becoming ever more important, given that the changing climate is increasing the frequency and duration of hot weather. Furthermore, the heat experienced in urban settings is amplified due to the heat island effect (United States Evironmental Protection Agency 2022). There is a lack of data showcasing potential associations between heat and injury outcomes in children, overall, or across categories of age, sex, race and ethnicity, and community-level factors (Ubiel *et al* 2022). In 2019, 7% of persons younger than 25 years were seen in emergency departments (EDs) in the US for unintentional injury, and 0.7% for intentional injury (Center for Disease Control and Prevention 2023). The majority of unintentional injuries within this population result from falls (28.1%), being struck or hit (20%), overexertion (9.1%), motor vehicle accidents (7.9%), and being cut or pierced (6.9%). Most intentional injuries are attributable to being struck or hit (55.7%), cut or pierced (15.8%), and poisoning (13.4%) (Center for Disease Control and Prevention 2023). Their associated cost of medical care and work loss were estimated to be $55 billion for unintentional injury and $3 billion for intentional injury. (Center for Disease Control and Prevention 2019). While environmental factors associated with injury remain underexplored, multiple factors are associated with this large burden of injury-related ED visits, such as age, ethnicity, and location (Center for Disease Control and Prevention 2019). With a substantial burden of injury morbidity among children and young adults, and evidence of a positive association between high ambient temperature and injury within pediatric populations, we aimed to examine these association within the New York City pediatric population, with attention to variation by outcome and individual-level susceptibility factors.

### 1.1. Potential mechanisms for the association between heat and intentional and unintentional injury

Injury is often categorized into two main groups: unintentional and intentional. Unintentional injury is defined as ‘injury or poisoning that is not inflicted by deliberate means’. Whereas, intentional injury includes assault cases (which includes sexual assault and legal intervention); meaning a confirmed or suspected injury from an act of violence where physical force by one or more persons is used with the intent of causing harm, injury or intentional poisoning by someone (Center for Disease Control and Prevention 2019). Potential mechanisms explaining the association between heat and injury vary by characteristics such as age, intent, type of injury, and pathway such as physiologic (i.e. sleep disruption) (Callaway *et al* 2005, Xu *et al* 2014, Im Kampe *et al* 2016, Runions *et al* 2019), sociologic (i.e. caregiver supervision, differences in risk taking by gender, gender roles) (Saluja *et al* 2004, Morrongiello and Matheis 2007, Archer 2009) and psychologic (i.e. mood disturbance) (Anderson 2001) mechanisms. When it is hot outside, exposed individuals may have impaired response abilities, which may in turn, result in unintentional injury (Ebi *et al* 2021). Some literature establishes an association between outdoor temperature and violent crime (Schinasi and Hamra 2017, Tiihonen *et al* 2017), which may lead to intentional injuries. More broadly, the sociologic literature suggests that intentional injury—which would include violent crimes that resulted in injury—may also be associated with warmer outdoor temperatures, potentially driven by more people being outside and having an opportunity to interact or have altercations (called routine activity theory) (Harp and Karnauskas 2018). As these mechanisms potentially drive intentional and unintentional injury differently and could be affected differently by warm outdoor temperatures, we examine intentional and unintentional injury cases separately.

We hypothesize high ambient temperatures are associated with higher risk of ED utilization for unintentional and intentional injuries in children and young adults, and especially among male adolescents and young adults than younger children and females of all ages. To test this hypothesis, we leveraged data from four NOAA meteorological stations in New York City linked with New York Statewide Planning and Research Cooperative System (SPARCS) dataset to estimate overall associations, and associations stratified by type of injury, sex, and age category. This is one of the first studies to explore relationships between warm season ambient temperature and intentional and unintentional injury among children and young adults.

## 2. Methods

### 2.1. Health data and study population

Data on all daily ED visits in New York City from 2005 to 2011 were extracted from the New York Statewide Planning and Research Cooperative System (SPARCS). SPARCS is a comprehensive all-payer administrative claims database containing over 95% of inpatient and ED visits in New York State (New York State Department of Health 2022). It contains patient-level data on demographic characteristics, diagnoses and treatments, services, and charges for each visit. We restricted our study population to ED visits that occurred during the typically warm months of May, June, July, August, and September between 2005 and 2011, as our goal was to study heat effects on injury ED visits. We focused on children and young adults between ages 0–25 years, to capture the risk of injury across the span of changing environments and risk factors throughout a child’s development into early adulthood.

### 2.2. Health outcomes

Injury-related ED visits were identified by selecting patients with International Classification of Diseases, Ninth Revision (ICD-9) External Cause of Injury Code (e.g. E-code). E-codes are assigned to patients who present to a healthcare provider with an injury and they are used to explain the circumstances of an injury by intent, mechanism, place of occurrence, and activity (National Center for Injury Prevention and Control 2009). We utilized E-codes to elucidate intent and mechanism of injury. We grouped the E-codes into 22 subtypes of injury (e.g. cut/pierce, fall, etc) that are the most common non-fatal injuries within the pediatric patient population in the U.S. in 2015 (Ballesteros *et al* 2018). The injury cases were then further categorized into two types: Unintentional and Intentional, defined using a CDC grouping framework based on E-codes (Center for Disease Control and Prevention 2018). The E-codes used to identify the injury outcomes analyzed are included in supplemental table S1.

We also obtained injury patients’ demographic data on race/ethnicity (white, non-Hispanic Black, Hispanic, Asian, and other), insurance/primary source of payment (commercial, Medicaid, self-pay, and other payment sources including worker’s compensation, Medicare, other federal program, BlueCross, CHAMPUS, and other non-federal program).

### 2.3. Meteorology data

We utilized data on daily minimum temperature (Tmin) averaged across four meteorological stations in the NYC area (JFK International Airport, LaGuardia Airport, Newark Liberty International Airport and Central Park) from the National Oceanic and Atmospheric Administration (NOAA) National Climate Data Center (NCDC) (NOAA n.d.). The data obtained from the four stations were averaged into one city-wide trend. Temperatures measured across these four locations were found to be highly correlated with small spatial variance, and the correlation were robust for all temperature metrics used (Sheffield *et al* 2018). We used minimum temperature (Tmin) because it is most representative of evening temperatures and nighttime (which would correspond to minimum daily temperatures) is when the urban heat island effect is most pronounced (Barnett *et al* 2010, Winquist *et al* 2016, Sheffield *et al* 2018, United States Evironmental Protection Agency 2022). The literature suggests that a loss of sleep has acute effects on mood, mental health, and susceptibility to aggression (Kamphuis *et al* 2012, Blackwelder *et al* 2021), which could also be associated with unintentional injury (Huang and Ihm 2021). In previous research, it has been found that Tmin is highly correlated with daily maximum temperature (*r* = 0.96) and mean temperature (*r* = 0.99) (Niu *et al* 2022). Relative humidity was calculated from mean temperature and dew point temperature following the standard NOAA equation using the R package *weathermetrics* (Anderson and Peng 2012).

### 2.4. Statistical analysis

We applied a case-crossover design to our injury dataset to study the association between daily Tmin and injury-related ED visits. We matched control days by case date, based on day of the week, month and year; generating 3 or 4 control days per case day (Janes *et al* 2005). This case-crossover approach inherently adjusts for individual time-independent factors. The control matching method adjusts for long-term trends, seasonality, and day-of-week (Maclure 1991).

We ran conditional logistic regression to estimate the effects of Tmin on the odds of injury-related ED visits and modeled the Tmin variable with distributed lag non-linear model (DLNM) functions. DLNM is a framework that allows us to simultaneously observe non-linear and lagged effects on an outcome with time-series exposure data (Gasparrini 2011). We modeled the non-linear effect of Tmin cumulatively over lag days 0–5 on injury-related ED visits. We chose this lag period based on our prior analyses of ambient temperature and ED visits, which explored varying lag structures and functional forms (Winquist *et al* 2016, Sheffield *et al* 2018, Niu *et al* 2023). These studies commonly examined a 6 d lag period (case day plus six lag days); prior results indicated significant effects of heat only on the case day (supplemental figure S5). Consistent with previous research, the lag-response curve for Tmin was modeled as a cubic spline with 3 degrees of freedom, to account for the non-linear relationship between Tmin and injury-related ED visits (Niu *et al* 2023).

The effect estimate was calculated as the cumulative odds ratios (ORs) over all lag days, representing the relative effect of an elevated temperature at 77°F, which corresponded to as the 95th percentile in our data to a reference temperature of 48°F. The range of Tmin through the study period was 43°F–86°F, with a mean of 65.6°F and a standard deviation of 7.9°F. The reference temperature was identified as the lowest temperature of minimum effect within the exposure-response model including all case days (unintentional and intentional injury and all ages combined) after 1st and 99th percentile temperatures were removed. This was calculated using the *findmin* function in R (Tobias *et al* 2017). The reference temperature was used in the subsequent stratified models.

We stratified by the following age groups: 0 years, 1–4 years, 5–9 years, 10–14 years, 15–19 years, and 20–25 years for the unintentional injury analysis but limited to only 15–19 and 20–25 for intentional analyses due to limited sample size in the younger age groups. We further stratified the age groups by sex to examine potential differences in effect estimates that may be driven by behavioral and sociological differences by age and sex (being a proxy for the social construct, gender).

Statistical analyses were done using SAS version 9.4 and R version 4.1.3 using the *dlnm* and *survival* package (Gasparrini 2011).

## 3. Results

In May through September in 2005–2011, there were a total of 572 535 injury-related ED visits in NYC. The largest proportions of injury were among male (60%), non-Hispanic Black (32%) and Hispanic (28%), and the primary source of payment was commercial insurance (63%) (table 1). The vast majority (91%) of the injury-related ED visits were categorized as unintentional injuries. A majority of the ED visits for injury were among males (60%) for both unintentional (60%) and intentional (68%) injury. While unintentional injuries are generally evenly distributed across the age groups, the age distribution among intentional injury patients is skewed to the older age groups, 10–14 years (13%), 15–19 years (38%), and 20–25 year olds (46%). Nearly half (45%) of intentional injuries occurred in people identified as non-Hispanic black, whereas the majority of unintentional injury is shared between people identified as white (21%) and non-Hispanic black (31%). Among intentional injury patients, the majority payment source is commercial insurance (44%), self-pay (39%) compared with unintentional injury patients where the most common payment source was commercial insurance (65.4%). The most prevalent unintentional injuries were falls, being struck, cuts/piercings, bites/stings and being an occupant in a motor vehicle accident (figure S2). The most prevalent intentional injury subtypes were being struck, cuts/piercings, fires/burns, other/human bites, and unknown/unspecified assault.

**Table 1.** Demographic characteristics for all NYC-resident children and young adults aged 0–25 years presenting in NYC metropolitan emergency departments for injury (unintentional and intentional) during the warm season (May-September), 2005–2011.

	Unintentional *n* = 522 343 (91%)	Intentional *n* = 50 192 (9%)	Total *n* = 572 535
Age			
0 years	18 453 (3.5%)	66 (0.1%)	18 514 (3.2%)
1–4 years	131 971 (25.3%)	422 (0.8%)	132 363 (23.1%)
5–9 years	99 636 (19.1%)	1382 (2.7%)	101 004 (17.6%)
10–14 years	82 898 (15.9%)	6545 (13.0%)	89 431 (15.6%)
15–19 years	79 180 (15.2%)	18 840 (37.5%)	98 004 (17.1%)
20–25 years	110 300 (21.1%)	22 937 (45.7%)	133 219 (23.3%)
Sex			
Female	210 780 (40.4%)	15 977 (31.8%)	226 757 (39.6%)
Male	311 563 (59.6%)	34 215 (68.2%)	345 778 (60.4%)
Race/ethnicity (missing = 5793)			
White	106 161 (20.5%)	5340 (10.8%)	111 501 (19.7%)
Non-Hispanic black	159 057 (30.7%)	22 439 (45.3%)	181 496 (32.0%)
Hispanic	148 030 (28.6%)	13 069 (26.4%)	161 099 (28.4%)
Asian	28 069 (5.4%)	1457 (2.9%)	29 526 (5.2%)
Other	75 871 (14.7%)	7249 (14.6%)	83 120 (14.7%)
Payment source (missing = 220 384)			
Commercial	209 849 (65.4%)	13 661 (43.9%)	223 510 (63.5%)
Medicaid	42 820 (13.3%)	4826 (15.5%)	47 646 (13.5%)
Self-pay	61 055 (19.0%)	12 229 (39.3%)	73 284 (20.8%)
Others	7324 (2.3%)	387 (1.2%)	7711 (2.2%)

### 3.1. Associations between daily minimum temperature and injury-related ED utilization by age groups

**Figure 1.**
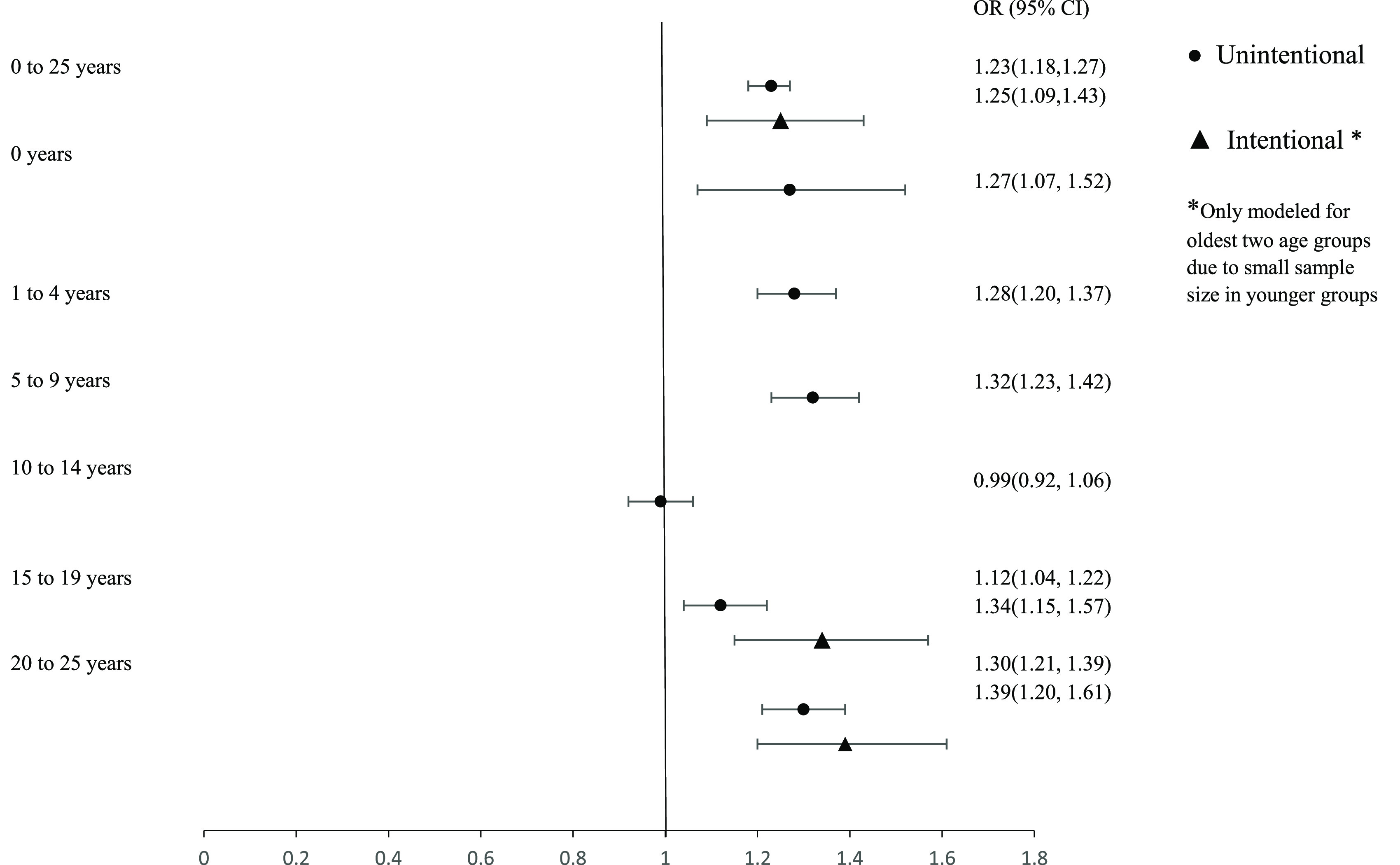
Cumulative odds ratio estimates of injury-related ED for children and young adults living in New York City, comparing odds of Unintentional and Intentional injury on 77 degree vs 48 degree days. Odds ratios are stratified by age and by type of injury (years 2005–2011).

**Figure 2.**
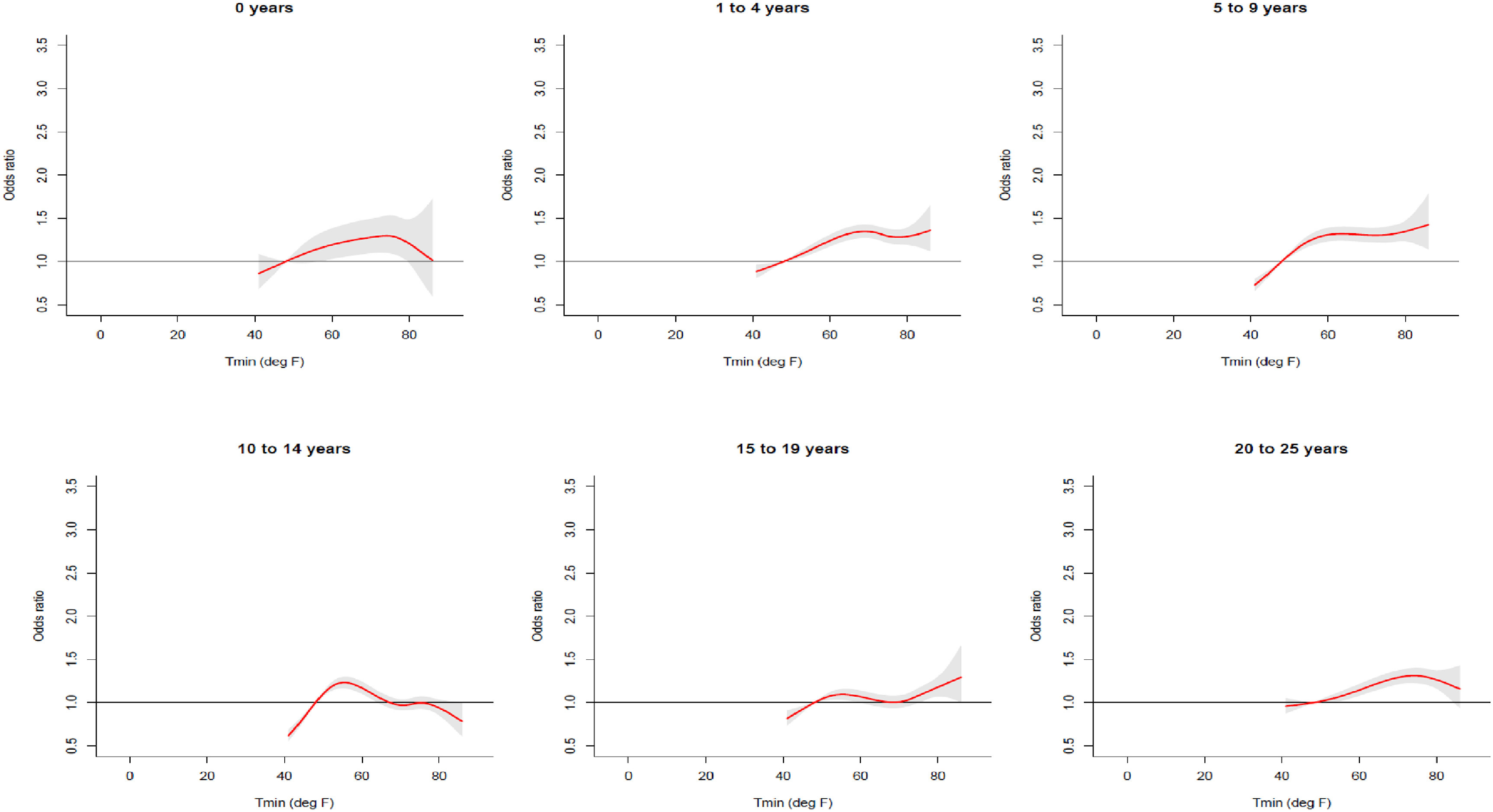
Cumulative odds of unintentional injury-related ED visits by Age Group, during May-September, 2005–11, among children and young adults aged 0–25 years living within New York City (adjusted for Relative Humidity).

The highest OR estimate of association between temperature and unintentional injury-related ED visits, is among 5–9 year olds (OR 1.32, 95% CI 1.23, 1.42). The highest OR for intentional injury-related ED visit, is among 20–25 year olds (OR 1.39, 95% CI 1.20, 1.61) (figure 1). The odds of unintentional injury is similar between all age groups except for 10–14 years (OR 0.99, 95% CI 0.92, 1.06) and 15–19 years (OR 1.12, 95% CI 1.04, 1.22). Odds for intentional injury varied by age group and was statistically significant (using a type one error threshold of 0.05) among 15–19 years olds (OR 1.34, 95% CI 1.15, 1.57) and 20–25 years old (OR 1.39, 95% %CI 1.20, 1.61). The odds of intentional injury varied by age group much more than unintentional injury, but exhibited a similar yet significant protective odds for 10–14 years (OR 0.79, 95% CI 0.63, 0.99). For unintentional injury-related visits, the shape of the exposure-outcome curve of cumulative lag days 0–5 varied by different age groups (figure 2). For children under the age of one and 20–25 year olds, the OR of unintentional injury increase linearly and decreases after reaching a moderate temperature, while the curves flatten out at moderate to high temperatures for 1–4 years, 5–9 years, and 15–19 years. The only exception was among children aged 10–14 years, where we see a slightly protective effect at moderate to high temperatures.

**Figure 3.**
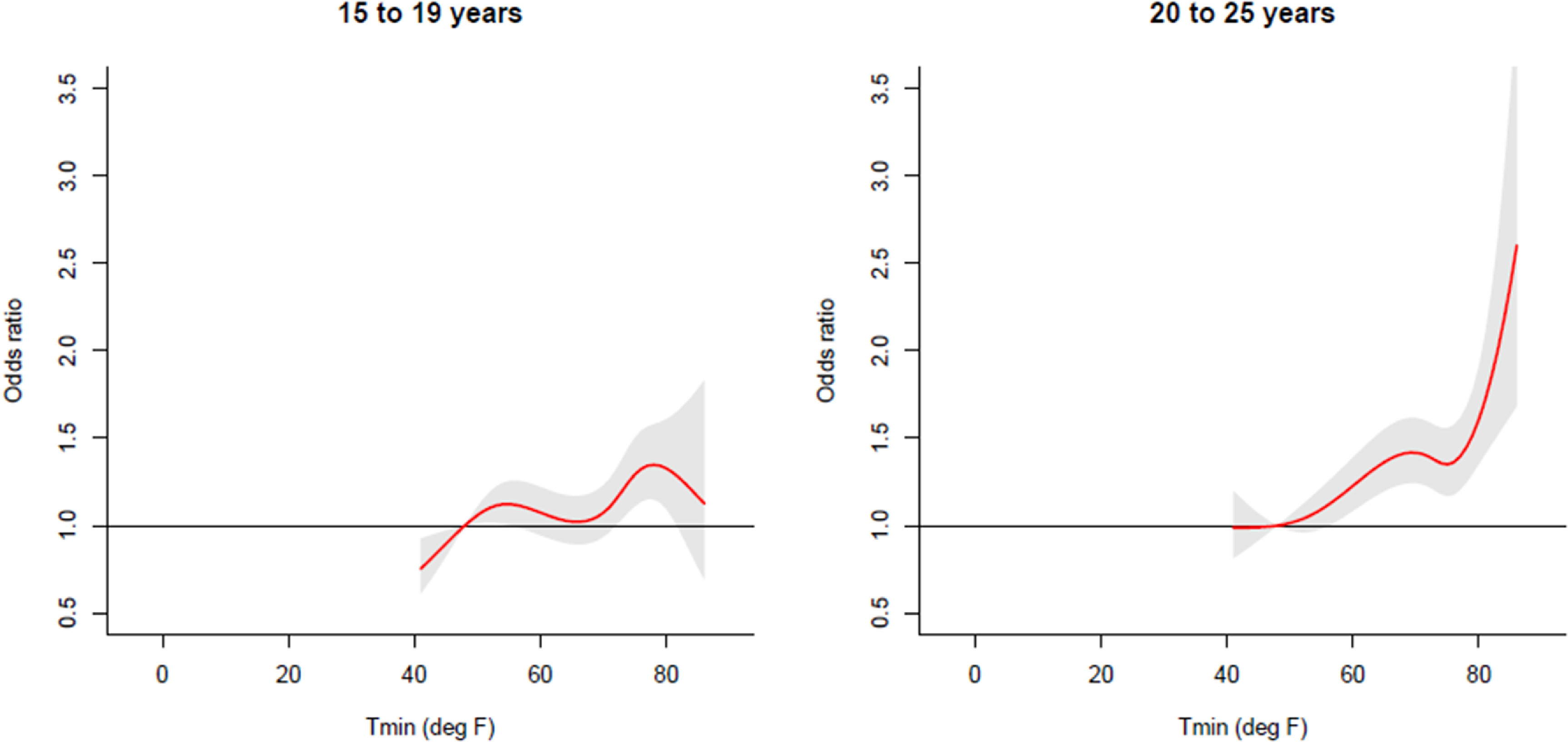
Cumulative odds of intentional injury-related ED visits by Age Group, during May-September, 2005–11, among children and young adults aged 15–25 years living within New York City (adjusted for Relative Humidity).

For intentional injury, the exposure-outcome curve was different between 15-19 years and 20–25 years (figure 3). For 15–19 year olds, the ORs fluctuate, where the slightly increase at moderate temperatures but decreases at high temperatures. For 20–25 years, the odds sharply increase at high temperature and continue to increase.

### 3.2. Stratified analysis by age x sex

**Figure 4.**
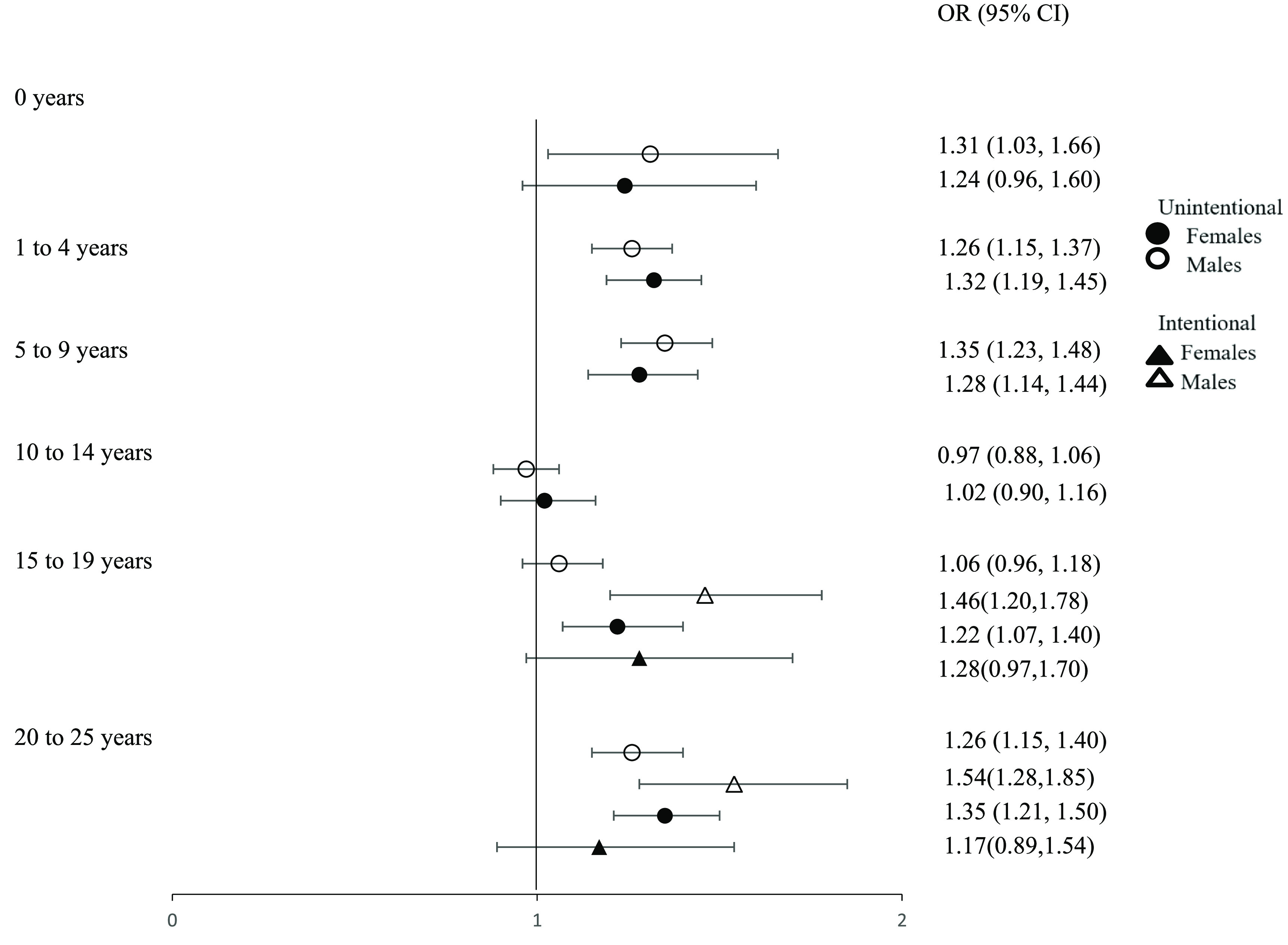
Cumulative odds ratio estimates of injury-related ED for children and young adults living in New York City, comparing odds of Unintentional and Intentional injury on 77 degree vs 48 degree days. Odds ratios are stratified by age, sex and by type of injury (years 2005–2011).

Age stratified estimates of association did not vary substantially by categories of sex (figure 4). Among males, the highest odds of an unintentional injury-related ED visit occurred among those aged 5–9 years (OR 1.35, 95% CI 1.23, 1.48) and among females, the highest risks were among those aged 15–19 years (OR 1.22, 95% CI 1.07, 1.40) 20–25 years (OR 1.35, 95% CI 1.21, 1.50). The highest odds of an intentional injury-related ED visit, among males are young adults aged 20–25 years (OR 1.54, 95% CI 1.28, 1.85) and among females, adolescents aged 15–19 years have the highest OR of intentional injury (figure 4).

When age groups are stratified by sex, the exposure-outcome curve for unintentional injury ED visit differ for 1–4 and 5–9 compared to models stratified only by age. (figure S3). Among 1–4 year olds, the odds for unintentional injury among males increase as temperatures rise while odds decrease for females. However, among 5–9 year olds, the odds of unintentional injury among males flatten at moderate to high temperatures and increases at high temperatures for females. Exposure-outcome curve trends for intentional injury ED visits stratified by age and sex are similar to trends stratified by age group only.

For intentional injury, Tmin-unintentional injury ED visit curves for both 15–19 and 20–25 year olds also differ when stratified by sex from the models stratified by age only (figure S4).

## 4. Discussion

We aimed to examine estimated associations between high ambient temperature and injury among children and young adults overall and according to categories of age and sex, and type of injury. The temperature-injury association we observed with unintentional injury ED visits were strongest among males age 5–9 years and among males age 20–25 years for intentional injury ED visits, suggesting vulnerability at these age groups. In summary, we have demonstrated approximately 30% higher odds of an ED visit for injury (unintentional and intentional across almost all age groups of young people) comparing some of the warmest (95th %) with the coolest temperatures during the warm season. Our results underscore the importance of considering variation in the health effects of heat, and consequences for healthcare utilization, given the growing impact of climate change. This research raises awareness of the multifaceted ways by which climate change and the urban climate impact children’s health and well-being.

We found the impact of heat differ by injury type and age group. Developmental literature suggests that risk taking increases as age increases (Morrongiello and Matheis 2007), however we see that younger age groups are at most risk to utilize ED services for unintentional injury in warmer weather than their older counterparts. These discrepancies may suggest the differences in the underlying mechanisms of unintentional injury, and the behavior pattern among different age groups. The most common unintentional injury subtype across all age groups during this study period are falls so the underlying mechanism may be related to an increase in activity and/or an effect on the child’s caregiver’s ability to supervise (Saluja *et al* 2004) Although we saw an overall higher risk of injury among males, we still observed some variation in the effect of high temperatures through the interaction of gender and age, where females had a higher risk of ED utilization for unintentional injury than males at older age groups. Literature on gender differences in risk taking and injury suggest that age, cognition, and anxiety are more predictive of risk-taking than gender (Morrongiello and Matheis 2007, Reniers *et al* 2016), but more specifically that anxiety did not have much of an effect on risk taking among females (Dorfman *et al* 2016). These may be factors which influence risk of injury between gender at different age groups and that may be interacting with the effects of heat.

While some studies suggest overall time outdoors may be similar across gender, there is variation by age and type of activity during the time outdoors (Larson *et al* 2011, Mullan 2018), particularly young females engage in more physical activity than young males when provided time to go outside (Kwon *et al* 2022). However, due to limitations with our data in characterizing enough intentional injury cases among younger children, we were not able to expand on potential mechanisms. Literature suggests that heat can impact aggressive behavior (Callaway *et al* 2005) and that there is sociological basis called the social role theory (Archer 2009) which influences males to perform physical aggression more than females. Following this logic and the evidence suggesting the impact of heat on crime through the routine activities theory ((Harp and Karnauskas 2018) as described above), the stronger heat-injury association among the 20–25 year olds, suggests that older males are at high risk of injury due to physical violence in hot weather.

### 4.1. Limitations

The generalizability of this study is limited in that we examined one city using an administrative dataset though these findings may be generalizable to NYC. Although our study leverages solely the temporal variation in minimum temperature at four NOAA monitors, we are examining a relatively small spatial region (within NYC city limits only), and demonstrated, in our previous work, that the spatial variance in temperature within our region is very small in comparison to the temporal variation and that varying temperature metrics are also very highly correlated (Sheffield *et al* 2018). Additionally, this study represents only an initial inquiry into heat and injury associations for children and young children. While we demonstrated an association, the relationship appears to vary by multiple parameters such as age groups, gender, and injury groupings—potentially due to limited power because of few very hot days and small sample sizes when we stratify the groups. The small sample size of subgroups also resulted in our inability to observe effects stratified by race/ethnicity and insurance/payment source, as well as younger age groups within the intentional injury group. Further, the complexity in characterizing injury resulting from violence adds to the challenge of this research. For example, a separate study found that temperature had a strong positive effect on frequency of violent crimes but little influence on physical injury of crime victims (Cruz *et al* 2020). Additionally, there could be biases among E-codes/billing codes used to define intentional injury and thus these findings should be interpreted with caution and not necessarily extrapolated as a proxy for violence-related injury. The data used for this study is over a decade old and subsequent studies should corroborate these findings with newer data where observed changes could be useful to inform subsequent public health interventions. Future directions for this work will include incorporation of spatiotemporal temperature metrics (Rowland *et al* 2021).

## 5. Conclusion

Subsequent work with more defined subcategories and sufficient sample size could explore race/ethnicity and socio-economic or other social disparities in the injury/heat association. We plan on expanding this research to more current years as data become available to us. This will allow us to compare the effects of heat on injury among children before and after the peak of the COVID-19 pandemic and changes in climate, elucidating differences in reporting and incidence of injury presented in the ED through the pandemic and the impact of heat as global warming continues.

Ultimately the intent of this type of research is to inform interventions. Hospital-based injury and violence prevention programs could consider heat events as a higher risk period especially for work with youth during warmer weather. Parents, caregivers, schools and childcare programs should prepare resources to ensure children and caregivers are protected against the effects of heat during extreme heat events to reduce preventable injuries. We suggest that local, city, and state government consider the vulnerability of children and young adults in their extreme heat mitigation strategies, which could also include highlighting activities, resources and tools community members can use to alleviate social and mental health stressors during warmer periods.

## Data Availability

The data cannot be made publicly available upon publication due to legal restrictions preventing unrestricted public distribution. The data that support the findings of this study are available upon reasonable request from the authors.
